# *N*-Phenyl­morpholine-4-carboxamide

**DOI:** 10.1107/S1600536810052207

**Published:** 2010-12-24

**Authors:** Shuang-Ming Meng, Ke-Wei Wang, Hai Xie, Yue-Qin Fan, Yong Guo

**Affiliations:** a College of Chemistry and Chemical Engineering, Shanxi Datong University, Datong 037009, People’s Republic of China

## Abstract

In the title compound, C_11_H_14_N_2_O_2_, the urea-type NC=ON moiety [planar to within 0.0002 (13) Å] is inclined to the phenyl ring by 42.88 (8) Å, and the morpholine ring has a chair conformation. In the crystal, inter­molecular N—H⋯O hydrogen bonds link the mol­ecules into infinite chains in [001].

## Related literature

For amides as functional groups in biologically relevant mol­ecules, see: Allen *et al.* (2010 ▶). For the synthesis of this and similar compounds, see: Montalbetti *et al.* (2005 ▶).
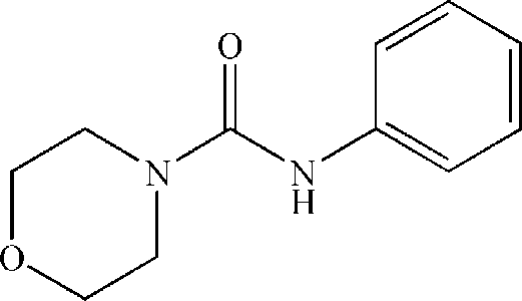


## Experimental

### 

#### Crystal data

C_11_H_14_N_2_O_2_
*M* *_r_* = 206.24
Monoclinic, 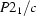

*a* = 8.0907 (10) Å
*b* = 15.754 (2) Å
*c* = 8.4529 (11) Å
β = 104.205 (2)°
*V* = 1044.5 (2) Å^3^
*Z* = 4
Mo *K*α radiation
μ = 0.09 mm^−1^
*T* = 293 K
0.29 × 0.21 × 0.19 mm

#### Data collection

Bruker SMART APEX CCD diffractometer
Absorption correction: multi-scan (*SADABS*; Bruker, 2001 ▶) *T*_min_ = 0.976, *T*_max_ = 0.981
5309 measured reflections
2056 independent reflections
1633 reflections with *I* > 2σ(*I*)
*R*_int_ = 0.016

#### Refinement

*R*[*F* ^2^ > 2σ(*F* ^2^)] = 0.040
*wR*(*F* ^2^) = 0.105
*S* = 1.04
2056 reflections
139 parameters
H atoms treated by a mixture of independent and constrained refinement
Δρ_max_ = 0.13 e Å^−3^
Δρ_min_ = −0.18 e Å^−3^

### 

Data collection: *SMART* (Bruker, 2007 ▶); cell refinement: *SAINT* (Bruker, 2007 ▶); data reduction: *SAINT*; program(s) used to solve structure: *SHELXS97* (Sheldrick, 2008 ▶); program(s) used to refine structure: *SHELXL97* (Sheldrick, 2008 ▶); molecular graphics: *SHELXTL-Plus* (Sheldrick, 2008 ▶); software used to prepare material for publication: *SHELXL97*.

## Supplementary Material

Crystal structure: contains datablocks global, I. DOI: 10.1107/S1600536810052207/su2236sup1.cif

Structure factors: contains datablocks I. DOI: 10.1107/S1600536810052207/su2236Isup2.hkl

Additional supplementary materials:  crystallographic information; 3D view; checkCIF report

## Figures and Tables

**Table 1:** Hydrogen-bond geometry (Å, °)

*D*—H⋯*A*	*D*—H	H⋯*A*	*D*⋯*A*	*D*—H⋯*A*
N1—H1*N*⋯O1^i^	0.844 (17)	2.130 (18)	2.9543 (16)	165.3 (16)

Symmetry code: (i) 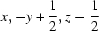.

**Fig. 1.**
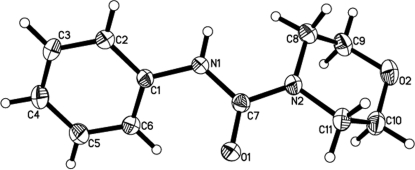
Molecular structure of the title molecule. Displacement ellipsoids are drawn at the 30% probability level.

**Fig. 2.**
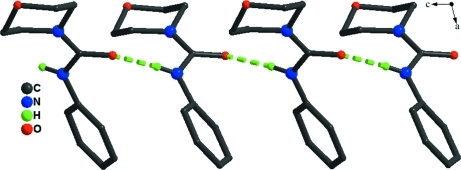
View along the b-axis of the one-dimensional polymeric chain of the title compound formed by hydrogen bonding (green dashed lines). H-atoms not involved in hydrogen bonding have been omitted for clarity.

## supplementary crystallographic information

### Comment

Amides are one of the most important and prolific functional groups found in biologically relevant molecules (Allen *et al.*, 2010), which lead to the synthesis of *N*-phenylmorpholine-4-carboxamide. In this study, this new acetamide derivative was prepared and its structure is presented herein.

In the title compound all the bond lengths and angles are within normal ranges. The molecule of the title compound is markedly non-planar (Fig. 1). The urea-type moiety [atoms N1,C7,O1,N2 - planar to within 0.0002 (13) Å] is inclined to the phenyl ring by 42.88 (8) Å. The morpholine ring has a chair conformation.

In the crystal, intermolecular N—H···O hydrogen bonds link the molecules into infinite one-dimensional chains propagagting in [001] (see Fig. 2 and Table 1).

### Experimental

The title compound was synthesized according to the literature procedure (Montalbetti *et al.*, 2005). To a solution of isocyanatobenzene (1.19 g, 10 mmol) and morpholine (0.87 ml, 10 mmol) in CH_2_Cl_2_ (25 ml) was added triethylamine (1.20 ml, 10 mmol) in one portion. The reaction mixture was stirred at room temperature for 3 h, and then poured into ice-water (100 ml) under stirring. The combined organic phase was washed with water (3 × 20 ml), dried over MgSO_4_, and filtered. Colourless single crystals were obtained by slow evaporation of the filtrate at room temperature.

### Refinement

The NH H-atom was located in a difference Fourier map and was refined with *U*_iso_(H) = 1.2*U*_eq_(N). The C-bound H-atoms were positioned geometrically and refined as riding: C—H = 0.93 Å (CH) and 0.97 Å (CH_2_) with *U*_iso_(H) = 1.2*U*_eq_(C).

### Crystal data

**Table:**

C_11_H_14_N_2_O_2_	*F*(000) = 440
*M**_r_* = 206.24	*D*_x_ = 1.312 Mg m^−^^3^
Monoclinic, *P*2_1_/*c*	Mo *K*α radiation, λ = 0.71073 Å
Hall symbol: -P 2ybc	Cell parameters from 5309 reflections
*a* = 8.0907 (10) Å	θ = 2.6–26.1°
*b* = 15.754 (2) Å	µ = 0.09 mm^−^^1^
*c* = 8.4529 (11) Å	*T* = 293 K
β = 104.205 (2)°	Block, colourless
*V* = 1044.5 (2) Å^3^	0.29 × 0.21 × 0.19 mm
*Z* = 4	

### Data collection

**Table:**

Bruker SMART APEX CCD diffractometer	2056 independent reflections
Radiation source: fine-focus sealed tube	1633 reflections with *I* > 2σ(*I*)
graphite	*R*_int_ = 0.016
ω scans	θ_max_ = 26.1°, θ_min_ = 2.6°
Absorption correction: multi-scan (*SADABS*; Bruker, 2001)	*h* = −8→9
*T*_min_ = 0.976, *T*_max_ = 0.981	*k* = −12→19
5309 measured reflections	*l* = −10→9

### Refinement

**Table:**

Refinement on *F*^2^	Primary atom site location: structure-invariant direct methods
Least-squares matrix: full	Secondary atom site location: difference Fourier map
*R*[*F*^2^ > 2σ(*F*^2^)] = 0.040	Hydrogen site location: inferred from neighbouring sites
*wR*(*F*^2^) = 0.105	H atoms treated by a mixture of independent and constrained refinement
*S* = 1.04	*w* = 1/[σ^2^(*F*_o_^2^) + (0.051*P*)^2^ + 0.1766*P*] where *P* = (*F*_o_^2^ + 2*F*_c_^2^)/3
2056 reflections	(Δ/σ)_max_ < 0.001
139 parameters	Δρ_max_ = 0.13 e Å^−^^3^
0 restraints	Δρ_min_ = −0.18 e Å^−^^3^

### Special details

**Table:**

Geometry. All e.s.d.'s (except the e.s.d. in the dihedral angle between two l.s. planes) are estimated using the full covariance matrix. The cell e.s.d.'s are taken into account individually in the estimation of e.s.d.'s in distances, angles and torsion angles; correlations between e.s.d.'s in cell parameters are only used when they are defined by crystal symmetry. An approximate (isotropic) treatment of cell e.s.d.'s is used for estimating e.s.d.'s involving l.s. planes.
Refinement. Refinement of *F*^2^ against ALL reflections. The weighted *R*-factor *wR* and goodness of fit *S* are based on *F*^2^, conventional *R*-factors *R* are based on *F*, with *F* set to zero for negative *F*^2^. The threshold expression of *F*^2^ > σ(*F*^2^) is used only for calculating *R*-factors(gt) *etc*. and is not relevant to the choice of reflections for refinement. *R*-factors based on *F*^2^ are statistically about twice as large as those based on *F*, and *R*- factors based on ALL data will be even larger.

### Fractional atomic coordinates and isotropic or equivalent isotropic displacement parameters (Å^2^)

**Table:**

	*x*	*y*	*z*	*U*_iso_*/*U*_eq_	
C1	0.32831 (18)	0.27851 (9)	0.53510 (15)	0.0363 (3)	
C6	0.21670 (19)	0.23577 (9)	0.60820 (17)	0.0423 (4)	
H006	0.2379	0.1796	0.6410	0.051*	
C2	0.2934 (2)	0.36162 (9)	0.48415 (18)	0.0446 (4)	
H007	0.3679	0.3909	0.4358	0.054*	
C7	0.56729 (17)	0.17667 (8)	0.59477 (16)	0.0354 (3)	
C11	0.79490 (19)	0.07168 (10)	0.61799 (19)	0.0466 (4)	
H00A	0.7747	0.0651	0.7258	0.056*	
H00B	0.9098	0.0932	0.6306	0.056*	
C5	0.0740 (2)	0.27684 (11)	0.6320 (2)	0.0533 (4)	
H010	0.0011	0.2486	0.6838	0.064*	
C9	0.6653 (2)	0.04917 (11)	0.2800 (2)	0.0532 (4)	
H01A	0.5540	0.0256	0.2783	0.064*	
H01B	0.6755	0.0528	0.1683	0.064*	
C8	0.6786 (2)	0.13675 (10)	0.35298 (18)	0.0515 (4)	
H01C	0.7849	0.1629	0.3450	0.062*	
H01D	0.5854	0.1716	0.2929	0.062*	
C10	0.7776 (2)	−0.01294 (10)	0.5331 (2)	0.0535 (4)	
H01E	0.8650	−0.0511	0.5929	0.064*	
H01F	0.6674	−0.0373	0.5327	0.064*	
C4	0.0387 (2)	0.35911 (12)	0.5799 (2)	0.0633 (5)	
H014	−0.0584	0.3862	0.5951	0.076*	
C3	0.1479 (2)	0.40080 (11)	0.5054 (2)	0.0587 (5)	
H015	0.1236	0.4561	0.4687	0.070*	
N2	0.67253 (16)	0.13193 (8)	0.52338 (14)	0.0450 (3)	
O1	0.55772 (13)	0.16176 (6)	0.73527 (11)	0.0438 (3)	
O2	0.79327 (14)	−0.00542 (7)	0.36958 (15)	0.0565 (3)	
N1	0.47270 (16)	0.23906 (8)	0.50123 (15)	0.0425 (3)	
H1N	0.505 (2)	0.2590 (11)	0.421 (2)	0.051*	

### Atomic displacement parameters (Å^2^)

**Table:**

	*U*^11^	*U*^22^	*U*^33^	*U*^12^	*U*^13^	*U*^23^
C1	0.0432 (8)	0.0365 (7)	0.0307 (6)	0.0074 (6)	0.0117 (6)	−0.0014 (5)
C6	0.0503 (9)	0.0360 (7)	0.0430 (8)	0.0046 (6)	0.0162 (7)	0.0020 (6)
C2	0.0550 (9)	0.0384 (8)	0.0453 (8)	0.0065 (7)	0.0213 (7)	0.0050 (6)
C7	0.0396 (7)	0.0314 (7)	0.0375 (7)	0.0000 (6)	0.0135 (6)	−0.0023 (6)
C11	0.0415 (8)	0.0472 (9)	0.0516 (9)	0.0114 (7)	0.0123 (7)	0.0019 (7)
C5	0.0504 (9)	0.0546 (10)	0.0616 (10)	0.0050 (8)	0.0265 (8)	0.0052 (8)
C9	0.0503 (9)	0.0595 (10)	0.0538 (9)	0.0068 (8)	0.0202 (7)	−0.0086 (8)
C8	0.0658 (11)	0.0506 (9)	0.0463 (8)	0.0153 (8)	0.0293 (8)	0.0033 (7)
C10	0.0486 (9)	0.0437 (9)	0.0701 (11)	0.0086 (7)	0.0185 (8)	0.0023 (8)
C4	0.0603 (11)	0.0606 (11)	0.0781 (12)	0.0253 (9)	0.0347 (9)	0.0106 (9)
C3	0.0698 (11)	0.0421 (9)	0.0711 (11)	0.0222 (8)	0.0306 (9)	0.0134 (8)
N2	0.0531 (8)	0.0446 (7)	0.0417 (7)	0.0159 (6)	0.0199 (6)	0.0024 (5)
O1	0.0544 (6)	0.0438 (6)	0.0369 (5)	0.0098 (5)	0.0182 (5)	0.0034 (4)
O2	0.0538 (7)	0.0500 (7)	0.0693 (8)	0.0108 (5)	0.0220 (6)	−0.0118 (6)
N1	0.0525 (8)	0.0407 (7)	0.0411 (7)	0.0138 (6)	0.0245 (6)	0.0091 (5)

### Geometric parameters (Å, °)

**Table:**

C1—C2	1.386 (2)	C5—H010	0.9300
C1—C6	1.387 (2)	C9—O2	1.414 (2)
C1—N1	1.4132 (18)	C9—C8	1.504 (2)
C6—C5	1.380 (2)	C9—H01A	0.9700
C6—H006	0.9300	C9—H01B	0.9700
C2—C3	1.379 (2)	C8—N2	1.4553 (18)
C2—H007	0.9300	C8—H01C	0.9700
C7—O1	1.2315 (16)	C8—H01D	0.9700
C7—N2	1.3559 (18)	C10—O2	1.424 (2)
C7—N1	1.3711 (18)	C10—H01E	0.9700
C11—N2	1.4601 (18)	C10—H01F	0.9700
C11—C10	1.504 (2)	C4—C3	1.372 (2)
C11—H00A	0.9700	C4—H014	0.9300
C11—H00B	0.9700	C3—H015	0.9300
C5—C4	1.376 (2)	N1—H1N	0.844 (17)
			
C2—C1—C6	119.46 (13)	H01A—C9—H01B	108.0
C2—C1—N1	117.91 (13)	N2—C8—C9	109.95 (13)
C6—C1—N1	122.52 (12)	N2—C8—H01C	109.7
C5—C6—C1	119.76 (14)	C9—C8—H01C	109.7
C5—C6—H006	120.1	N2—C8—H01D	109.7
C1—C6—H006	120.1	C9—C8—H01D	109.7
C3—C2—C1	119.86 (15)	H01C—C8—H01D	108.2
C3—C2—H007	120.1	O2—C10—C11	111.67 (13)
C1—C2—H007	120.1	O2—C10—H01E	109.3
O1—C7—N2	121.67 (13)	C11—C10—H01E	109.3
O1—C7—N1	122.31 (12)	O2—C10—H01F	109.3
N2—C7—N1	116.02 (12)	C11—C10—H01F	109.3
N2—C11—C10	110.11 (13)	H01E—C10—H01F	107.9
N2—C11—H00A	109.6	C3—C4—C5	119.41 (15)
C10—C11—H00A	109.6	C3—C4—H014	120.3
N2—C11—H00B	109.6	C5—C4—H014	120.3
C10—C11—H00B	109.6	C4—C3—C2	120.78 (15)
H00A—C11—H00B	108.2	C4—C3—H015	119.6
C4—C5—C6	120.69 (15)	C2—C3—H015	119.6
C4—C5—H010	119.7	C7—N2—C8	126.22 (12)
C6—C5—H010	119.7	C7—N2—C11	120.64 (12)
O2—C9—C8	111.64 (14)	C8—N2—C11	113.14 (12)
O2—C9—H01A	109.3	C9—O2—C10	110.01 (11)
C8—C9—H01A	109.3	C7—N1—C1	124.79 (12)
O2—C9—H01B	109.3	C7—N1—H1N	119.4 (12)
C8—C9—H01B	109.3	C1—N1—H1N	115.7 (12)
			
C2—C1—C6—C5	−1.2 (2)	O1—C7—N2—C11	−8.0 (2)
N1—C1—C6—C5	−177.35 (13)	N1—C7—N2—C11	172.05 (13)
C6—C1—C2—C3	−0.5 (2)	C9—C8—N2—C7	−128.09 (16)
N1—C1—C2—C3	175.89 (14)	C9—C8—N2—C11	51.77 (18)
C1—C6—C5—C4	1.8 (2)	C10—C11—N2—C7	128.49 (15)
O2—C9—C8—N2	−55.97 (18)	C10—C11—N2—C8	−51.38 (18)
N2—C11—C10—O2	54.81 (17)	C8—C9—O2—C10	60.27 (17)
C6—C5—C4—C3	−0.8 (3)	C11—C10—O2—C9	−59.74 (17)
C5—C4—C3—C2	−0.9 (3)	O1—C7—N1—C1	−16.0 (2)
C1—C2—C3—C4	1.5 (3)	N2—C7—N1—C1	163.93 (13)
O1—C7—N2—C8	171.82 (14)	C2—C1—N1—C7	150.53 (14)
N1—C7—N2—C8	−8.1 (2)	C6—C1—N1—C7	−33.2 (2)

### Hydrogen-bond geometry (Å, °)

**Table:**

*D*—H···*A*	*D*—H	H···*A*	*D*···*A*	*D*—H···*A*
N1—H1N···O1^i^	0.844 (17)	2.130 (18)	2.9543 (16)	165.3 (16)

Symmetry codes: (i) *x*, −*y*+1/2, *z*−1/2.

## References

[bb1] Allen, C. L., Burel, C. & Williams, J. M. J. (2010). *Tetrahedron Lett.* **20**, 2724–2726.

[bb2] Bruker (2001). *SADABS* Bruker AXS Inc., Madison, Wisconsin, USA.

[bb3] Bruker (2007). *SMART* and *SAINT* Bruker AXS Inc., Madison, Wisconsin, USA.

[bb4] Montalbetti, C. & Falque, V. (2005). *Tetrahedron Lett.* **61**, 10827–10852.

[bb5] Sheldrick, G. M. (2008). *Acta Cryst.* A**64**, 112–122. 10.1107/S0108767307043930 18156677

